# Cloning, Expression, and Characterization of a New Glycosaminoglycan Lyase from *Microbacterium* sp. H14

**DOI:** 10.3390/md17120681

**Published:** 2019-12-02

**Authors:** Junhao Sun, Xu Han, Guanrui Song, Qianhong Gong, Wengong Yu

**Affiliations:** 1 School of Medicine and Pharmacy, Ocean University of China, 5 Yushan Road, Qingdao 266003, China; sunjunhao@stu.ouc.edu.cn (J.S.); songguanrui@stu.ouc.edu.cn (G.S.); 2 Laboratory for Marine Drugs and Bioproducts, Qingdao National Laboratory for Marine Science and Technology, 1 Wenhai Road, Qingdao 266237, China; 3 Provincial Key Laboratory of Glycoscience and Glycotechnology, Ocean University of China, 5 Yushan Road, Qingdao 266003, China

**Keywords:** glycosaminoglycan lyase, oligosaccharide, characterization

## Abstract

Glycosaminoglycan (GAG) lyase is an effective tool for the structural and functional studies of glycosaminoglycans and preparation of functional oligosaccharides. A new GAG lyase from *Microbacterium* sp. H14 was cloned, expressed, purified, and characterized, with a molecular weight of approximately 85.9 kDa. The deduced lyase HCLaseM belonged to the polysaccharide lyase (PL) family 8. Based on the phylogenetic tree, HCLaseM could not be classified into the existing three subfamilies of this family. HCLaseM showed almost the same enzyme activity towards hyaluronan (HA), chondroitin sulfate A (CS-A), CS-B, CS-C, and CS-D, which was different from reported GAG lyases. HCLaseM exhibited the highest activities to both HA and CS-A at its optimal temperature (35 °C) and pH (pH 7.0). HCLaseM was stable in the range of pH 5.0–8.0 and temperature below 30 °C. The enzyme activity was independent of divalent metal ions and was not obviously affected by most metal ions. HCLaseM is an endo-type enzyme yielding unsaturated disaccharides as the end products. The facilitated diffusion effect of HCLaseM is dose-dependent in animal experiments. These properties make it a candidate for further basic research and application.

## 1. Introduction

Hyaluronan (HA) and chondroitin sulfate/dermatan sulfate (CS/DS) belong to the class of negatively charged linear heteropolysaccharides named glycosaminoglycans (GAGs) [1,2], which are widely distributed on the surface of cells and connective tissue of animals. Enzymes degrading GAGs are associated with the regulation of various cellular processes, such as proliferation, differentiation, migration, and adhesion [3,4]. GAG-based therapeutic agents have important application prospects. According the Carbohydrate-Active enZYmes (CAZy) database, glycosaminoglycan lyases are classified into the enzymes of PL-8, PL-16, PL-29 and other families. Compared to other families, the enzymes of the PL-8 family can degrade more types of substrates. The enzymes of the PL-8 family are an important component of the GAG lyases, which can be divided into three subfamilies by phylogenetic analysis. Hyalurorate lyase (HAase), which mainly degrades HA, belongs to the first subfamily. Chondroitin ABC lyases (ChSase ABC) and chondroitin AC lyases (ChSase AC), which mainly degrade chondroitin sulfate, belong to the second and third subfamilies, respectively.

Different molar masses of HA fragments produced by enzymatic degradation perform different biological functions, such as induction of endothelial cell differentiation [5], promotion of angiogenesis [6], stimulation of collagen production, and proliferation of fibroblasts [7], and have attracted widespread attention. CS is mainly used to treat inflammation and osteoarthritis. It is not easily absorbed and may impair therapeutic effect because of its large molecular weight. The low molecular weight chondroitin sulfate (LMWCS) produced by enzymatic degradation is more easily absorbed and exerts therapeutic effects [8]. LMWCS might prevent and treat Alzheimer's disease through the blood–brain barrier and exert neuroprotective effects [9]. Chemical degradation can also produce oligosaccharides, but may alter their structure. Enzymatic approaches are used because of their specificity, and enzymatic degradation of polysaccharide is environmentally friendly [10].

GAG lyases degrade substrates such as HA and CS, which are effective tools for preparing bioactive oligosaccharide and studying the structure–function relationship of polysaccharides. GAG lyases have broad application prospects in the field of medicine because GAGs are widely distributed in the human body, which can treat lumbar disc herniation, spinal injury, and tumors [11,12]. Hyaluronidase is widely used as a drug diffusion agent in clinic [13]. Subcutaneous administration is a safe and convenient method of administration. However, the subcutaneous space has a complex three-dimensional extracellular matrix (ECM). The main filling of ECM is hyaluronan, which hinders the diffusion of drugs for subcutaneous administration [14]. Hyaluronidase can degrade hyaluronan in the subcutaneous space, improve tissue permeability, and promote drug diffusion and absorption. The currently commercial hyaluronidase is mainly prepared by extracting bovine testicular tissue. These enzymes are low in purity and high in price, limiting their use in pharmaceutical and biochemical engineering.

The marine environment contains abundant enzyme resources. The search for novel polysaccharide lyases is essential for academic research and applications. In our previous work, strain *Microbacterium* sp. H14 was isolated from offshore of Qingdao, which degraded HA and CS. In this study, the HA and CS lyase (HCLaseM) was cloned, expressed, purified, and characterized. Results show that HCLaseM has almost the same enzyme activity for HA and various CSs and may be an effective tool for structural analysis of HA and CS and preparation of oligosaccharides. HCLaseM has the potential to become a good drug diffusion agent.

## 2. Results

### 2.1. Cloning and Sequence Analysis of HCLaseM Gene

*Microbacterium* sp. H14 was isolated from offshore of Qingdao, which degraded HA and CS. Only one gene (GenBank MN400984.1) encoding GAG lyase was found in *Microbacterium* sp. H14 by genome sequencing analysis, which consists of an open reading frame of 2415 bp encoding 804 amino acids. The theoretical molecular weight of the deduced protein is 85.9 KDa and the isoelectric point is 5.79. The results of the NCBI sequence alignment showed that HCLaseM was a new enzyme of the PL-8 family. The characterized enzymes with the highest similarity to HCLaseM are chondroitin lyase (GenBank ANC28180.1) from *Arthrobacter* sp. GAG (45%) [15], chondroitin lyase (AFM38168.1) from *Arthrobacter* sp. MAT3885 (44%), and chondroitin lyase (PDB 1RWA_A) from *Arthrobacter aurescens* (43%) [16,17]. These enzymes are chondroitin sulfate AC lyases and cannot degrade CS-B. Chondroitin lyase (GenBank ANC28180.1) from *Arthrobacter* sp. GAG and chondroitin lyase (PDB 1RWA_A) from *Paenarthrobacter aurescens* are exogenous enzymes. However, HCLaseM is an endo-type enzyme and degrades CS-B. HCLaseM showed extensive substrate specificity. The relatively low similarity makes HCLaseM a more interesting and unique enzyme. 

**Figure 1:**
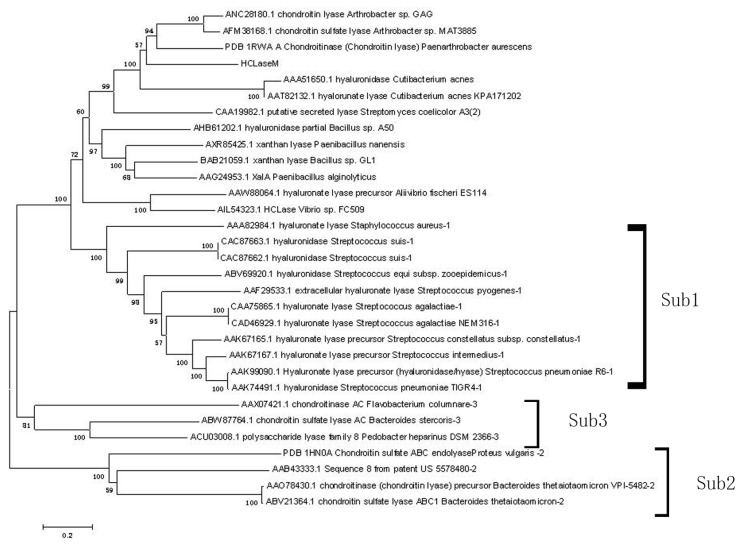
Phylogenetic analysis of HCLaseM and characterized enzymes of the PL-8 family. The phylogenetic analysis was performed using the neighbor joining method in MEGA 5.1.

A phylogenetic tree was constructed for chondroitin lyase of *Arthrobacter* sp. GAG, HCLaseM, and other characterized enzymes of the PL-8 family from the Carbohydrate-Active enZYmes (CAZy) database (Figure 1). Eleven enzymes were included in the first subfamily, four enzymes were included in the second subfamily, and three enzymes were included into the third subfamily. HCLaseM and other enzymes were not included into the existing three subfamilies. HCLaseM formed a distinct group with chondroitin lyase (PDB: 1RWA A), chondroitin lyase (AFM38168.1), and chondroitin lyase (ANC28180.1). The result show that based on the phylogenetic tree, HCLaseM cannot be classified into the existing three subfamilies of this family.

### 2.2. Purification and Biochemical Characterization of HCLaseM 

**Figure 2:**
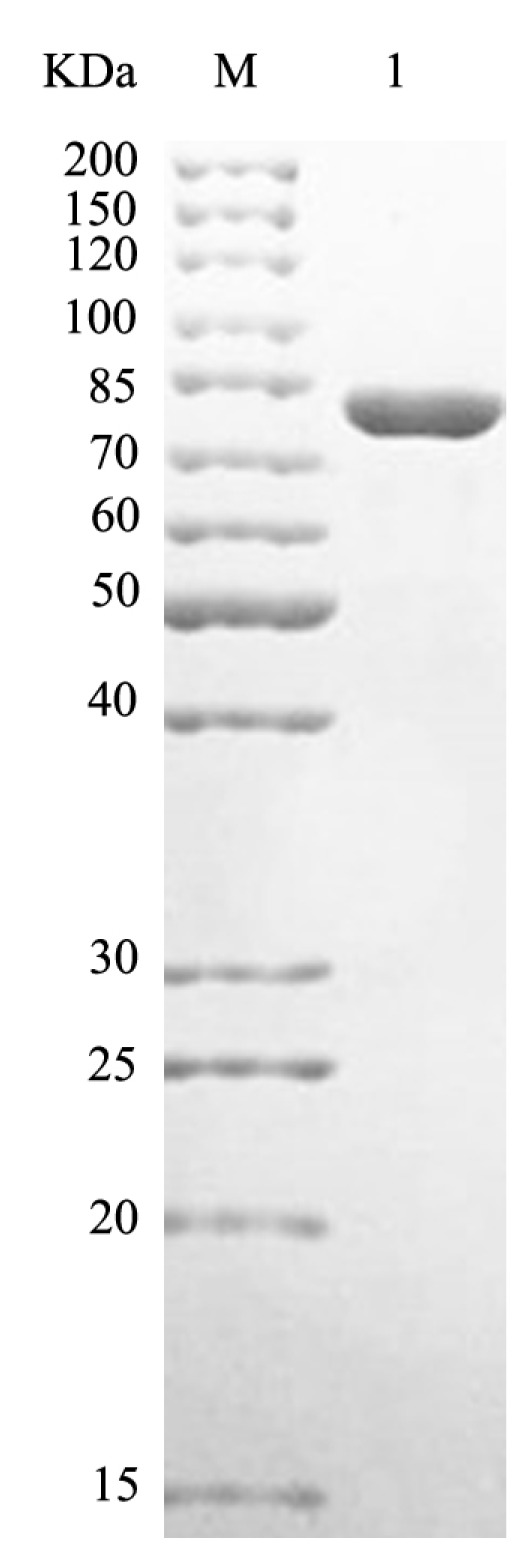
SDS-PAGE of HCLaseM. Lane M, protein marker of molecular mass; Lane 1, purified HCLaseM.

The recombinant plasmid was successfully constructed and expressed in *Escherichia coli* BL21 (DE3). The recombinant protein was purified by Ni-Sepharose column, and SDS-PAGE showed that HCLaseM was successfully purified with an estimated molecular weight of 85 kDa (Figure 2), which was consistent with the expected size. Using HA as a substrate, the specific activity of HCLaseM was 278.3 U/mg, and the recovery was 55.9%; 15.4 mg of purified HCLaseM was obtained from 1 L of bacterial culture (Table 1).

**Figure 3:**
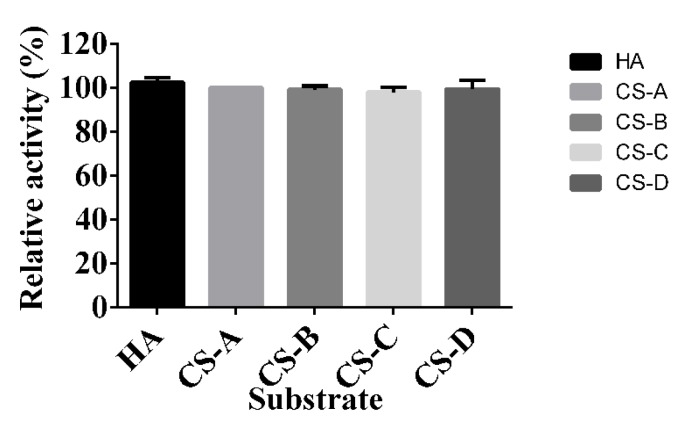
Substrate specificity of HCLaseM; 0.2% (*w*/*v*) of HA, CS-A, CS-B, CS-C, and CS-D were used as substrates. The relative activity of 100% was CS-A determined at optimal pH and temperature.

Five glycosaminoglycans were used as substrates to detect the specificity of HCLaseM. The relative activity of HCLaseM to CS-A, CS-B, CS-C, CS-D, and HA was 100%, 99%, 98%, 99%, and 102% (Figure 3), respectively. The V_max_ of HCLaseM against HA and CS-A was 0.0246 ± 0.0003 and 0.0264 ± 0.0002 μM/min (Table 2), respectively. The *K*_m_ of HCLaseM against HA and CS-A was 0.419 ± 0.019 and 0.478 ± 0.015 mg/mL, respectively. HCLaseM showed extensive substrate specificity and almost the same enzyme activity towards HA, CS-A, CS-B, CS-C, and CS-D, which was different from other conventional hyaluronidases and chondroitinase (Table 3). Both the relative activity (Figure 3) and *V*_max_ showed that HCLaseM was similarly active towards HA and CS-A, which indicates that it possessed similarly affinity towards HA and CS-A.

**Figure 4:**
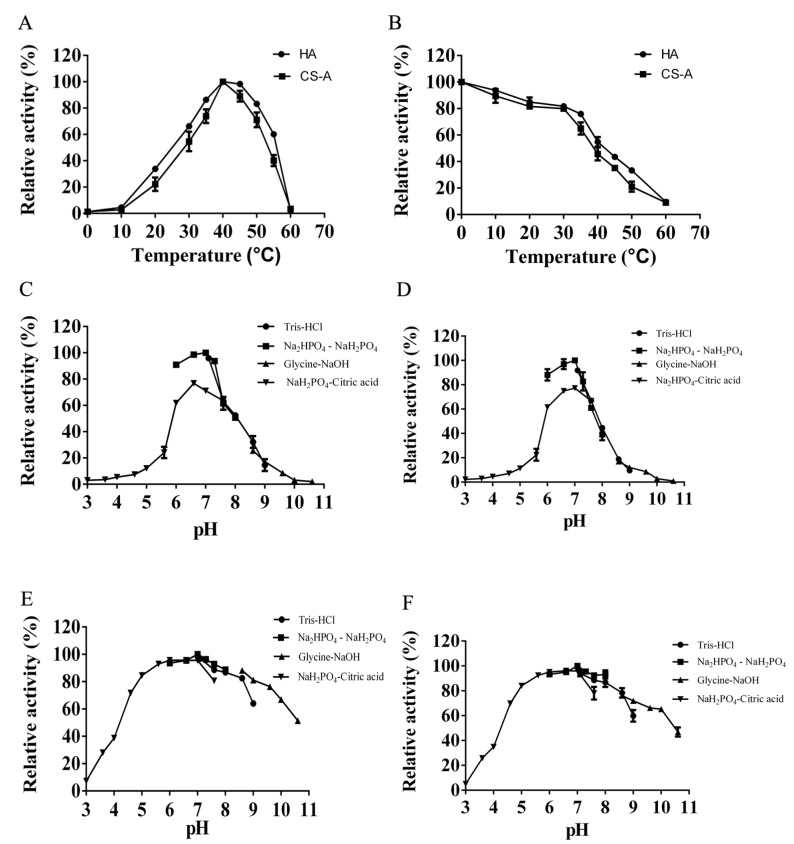
Effects of temperature and pH on HCLaseM. (**A**) Optimal temperature of HCLaseM. (**B**) The thermostability of HCLaseM. (**C**) Optimal pH of HCLaseM when using HA as a substrate. (**D**) Optimal pH of HCLaseM when using CS-A as a substrate. (**E**) The pH stability of HCLaseM when using HA as a substrate. (**F**) The pH stability of HCLaseM when using CS-A as a substrate. The relative activity of 100% was determined at optimal pH and temperature. Experiments were conducted three times and error bars represent standard deviations.

As the temperature increased, the enzyme activity of HCLaseM gradually increased at 0–40 °C (Figure 4A). The optimum temperature for the activity of HCLaseM towards both HA and CS-A was 40 °C. The HA/CS-degrading activity was stable below 30 °C, and remained about 50% after incubation at 40 °C for 60 min (Figure 4B). HCLaseM showed the highest activity at pH 7.0 (Figure 4C, D). HCLaseM retained about 70% of activity at pH 5.0–9.0, and retained about 90% of activity at pH 6.0–8.0 after incubation for 12 h (Figure 4E,F). These properties were beneficial to the storage of the enzyme. When HCLaseM was incubated in Glycine–NaOH buffer, the residual activity of CS-A was generally slightly lower than that of HA. Compared with the HA degradation activity of HCLaseM, the CS degradation activity is less tolerant to the alkaline environment.

**Figure 5:**
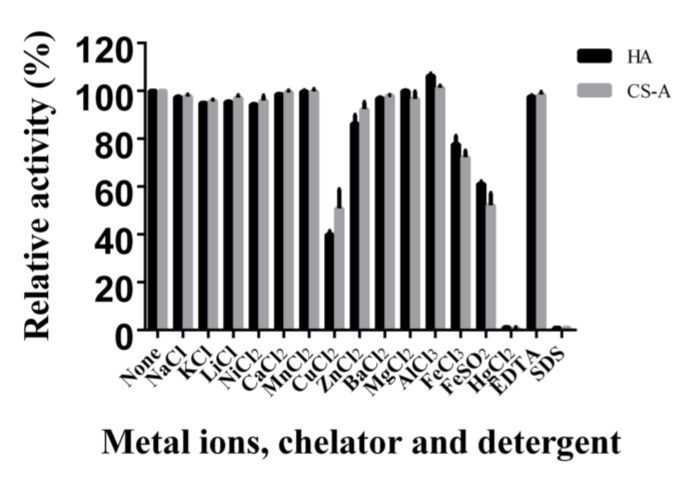
Effects of metal ions, chelators, and detergents on HCLaseM. The activities of HCLaseM against HA and CS-A were measured in 20 mM Tris-HCl (pH 7.0) buffer containing 1 mM concentration of various metal ions, chelators, and detergents. The relative activity of 100% was determined in the buffer without metal ions, chelators, and detergents.

The effect of various metal ions, chelators, and detergents on HCLaseM was examined. The activity of HCLaseM was strongly inhibited by Hg^2+^, Fe^2+^, Cu^2+^ and SDS, and slightly promoted by Al^3+^ (Figure 5). Except for Hg^2+^, Fe^2+^ and Cu^2+^, most metal ions did not significantly affect the activity of HCLaseM. EDTA as a metal ion chelating agent hardly reduced the activity of HCLaseM, which was different from previous reports that EDTA reduced enzyme activity [24]. 

### 2.3. Degradation Pattern and End Production of HCLaseM

**Figure 6:**
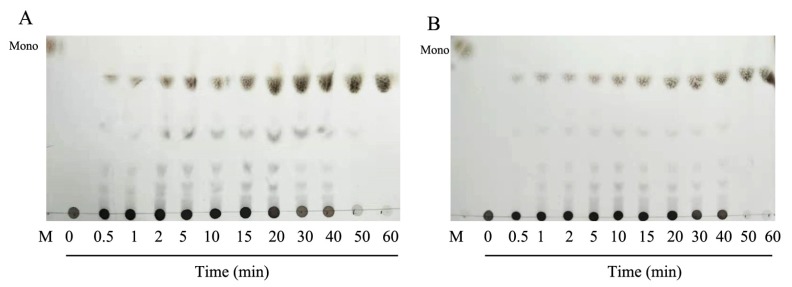
The reaction pattern of HCLaseM. The reactions were conducted at 40 °C using 0.2% (*w*/*v*) sodium HA and CS-A in 20 mM Na_2_HPO_4_–NaH_2_PO_4_ (pH 7.0) buffer. (**A**) The time course of HA degradation by HCLaseM was was determined by TLC. (**B**) The time course of CS-A degradation by HCLaseM was determined by TLC. Lane M, the purified monomeric sugar: *N*-acetyl-d-(+)-glucosamine.

**Figure 7:**
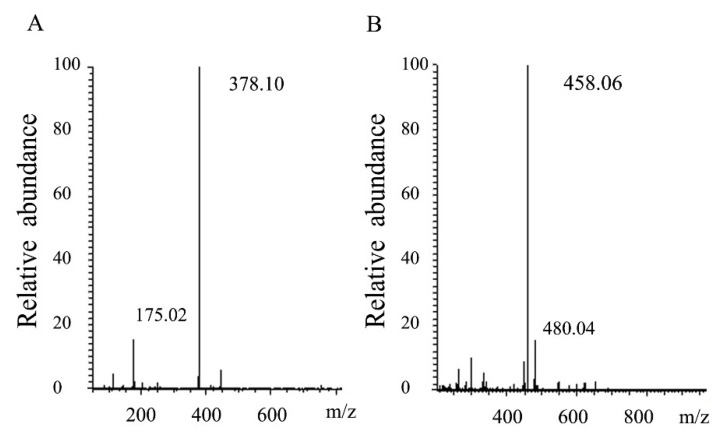
Analysis of end products of HCLaseM. (**A**) ESI–MS analysis of end products of HA. (**B**) ESI–MS analysis of end products of CS-A.

Thin layer chromatography was used to analyze the mode of degradation of HCLaseM, and the time course of the reaction products was analyzed by using hyaluronan as a substrate. It was found that a small amount of hyaluronan oligosaccharide with low molecular weight was present at the beginning of the reaction, and low molecular weight oligosaccharides gradually increased as the reaction proceeded (Figure 6A). The time course of the reaction product of CS-A (Figure 6B) was also similar, and the pattern of the oligosaccharide product suggests that HCLaseM may degrade substrates in an endolytic manner. The end-product was separated by Superdex peptide 10/300 GL gel filtration column and subjected to ESI–MS analysis. The main peaks of the final products of HA and CS-A were 378.10 *m*/*z* (Figure 7A) and 458.06 *m*/*z* (Figure 7B), respectively. The molecular weights are consistent with the unsaturated disaccharides of HA and CS-A, respectively. These results demonstrate that HCLaseM was an endo-type lyase, which could completely degrade HA and CS-A to unsaturated disaccharides through a β-elimination mechanism.

### 2.4. Effect of Hyaluronidase on the Diffusion of Trypan Blue

**Figure 8:**
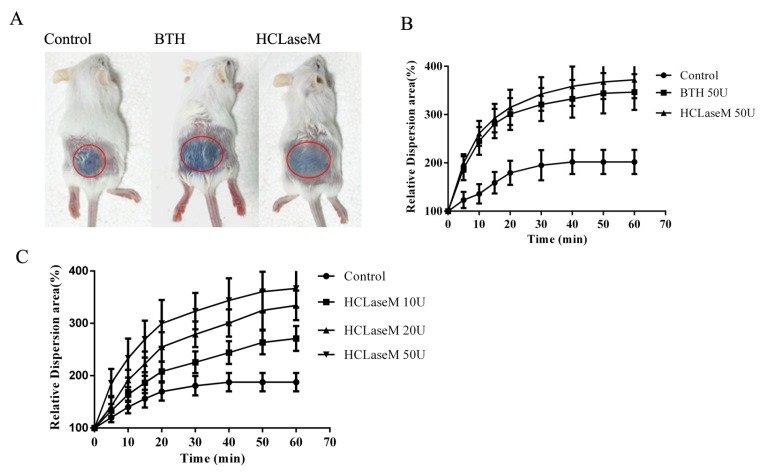
Diffusion properties of trypan blue with hyaluronidase co-administration in dermis. (**A**) Trypan blue diffusion 60 min after combined injection with PB, BTH, and HCLaseM. (**B**) HCLaseM and BTH (50 U) was co-injected with trypan blue in mice. (**C**) HCLaseM (10, 20 and 50 U) was co-injected with trypan blue in mice. The relative diffusion area of each group at 0 min is 100%. Error bars represent standard deviations.

The ability of hyaluronidase to promote diffusion can be determined by the increased diffusion area of trypan blue after co-administration in the skin [25]. The diffusion experiment of trypan blue characterizes the facilitated diffusion effect of hyaluronidase in a short period. Hyaluronidase from bovine testes (BTH) was used as a drug diffusion agent in clinic. The diffusion area of trypan blue in the experimental group was significantly higher than that in the control group (Figure 8A,B). The same dose of BTH and HCLaseM showed similar diffusion effects. This suggests that HCLaseM and BTH are equally effective in digesting hyaluronan in skin tissue in a short period. The potency of HCLaseM is even slightly higher than that of BTH, probably because HCLaseM has considerable activity to degrade chondroitin sulfate. The diffusion area of trypan blue increased with the increase of HCLaseM dose (Figure 8C). This suggests that the facilitated diffusion effect of HCLaseM is dose-dependent. 

## 3. Discussion

**Figure 9:**
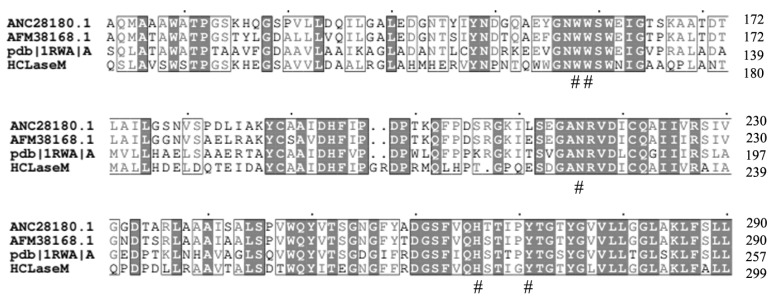
Comparison of the partial amino acid sequence of HCLaseM with chondroitin lyase (GenBank ANC28180.1) from *Arthrobacter* sp. GAG (45%), chondroitin lyase (AFM38168.1) from *Arthrobacter* sp. MAT3885 (44%), and chondroitin lyase (PDB 1RWA_A) from *Paenarthrobacter aurescens* (43%). Identical amino acids are shaded black. # Conservative amino acids.

HCLaseM contains three conserved catalytic residues (N225, H275, and Y284 numbering in HCLaseM; Figure 9), which is consistent with previous studies that Tyr residue acted as a general base for the abstraction of the proton from the C5 position of the glucuronic acid, while the Asn and His residues neutralized the charge on the glucuronic acid group [17]. Since the activity of HCLaseM is independent of divalent ions, the action of HCLaseM may utilize the catalytic mechanism of Tyr–His, rather than Arg/Lys acting as a Bronsted base and acid [21].

Aromatic patch (W371, W372, F423 in *Streptococcus* HL) and negative patch (E468, D478, T480 in *Streptococcus* HL) have been reported in *Streptococcus* HL [26,27]. These amino acids anchor the substrate chain into a degradation site [28]. HCLaseM has several conserved amino acids associated with substrate interactions. Aromatic patches were also conserved in all enzymes (W166 and W167 numbering in HCLaseM; Figure 9). The phenylalanine residue in the aromatic patch is not present in HCLaseM, which eliminates the structural conflict between HCLaseM and 4-sulfated CS. This might allow HCLaseM to degrade all kinds of sulfated CS. A clearly negative patch was not found in HCLaseM, which suggests that HCLaseM has a unique negative patch consisting of other amino acid residues. However, the precise mechanism of action of HCLaseM's broad substrate specificity has not been determined.

The enzymes of the PL-8 family can degrade many types of glycosaminoglycans and can be divided into three subfamilies by phylogenetic analysis. Hyaluronidase typically degrades HA and is relatively less active against CS. This type of hyaluronidase is generally the first subfamily of the PL-8 family, such as hyaluronidase from *Streptococcus suis* [29]. Chondroitin ABC lyase mainly degrades CS-A, CS-B, and CS-C. The relative enzymatic activity on HA is generally low. This type of lyase is generally the second subfamily, such as chondroitin ABC lyase from *B. thetaiotaomicron* with the activity towards HA at 10–30% of CS [30]. Chondroitin AC lyase mainly degrades CS-A and CS-C, and has certain activity on HA. This type of enzyme is generally the third subfamily, such as chondroitin AC lyase from *Bacteroides stercoris* with the activity towards HA was 67% of CS-A [18]. The evolution of original chondroitinase into later hyaluronidase plausibly explains this phenomenon. There are also some enzymes that are not included into three subfamilies, such as HCLase from *Vibrio* sp. FC509 [21]. HCLase has the highest activity towards HA, and the activity towards various CSs was relatively high (36–73% of hyaluronidase activity). Most enzymes have limitations in degrading HA and various CSs. HCLaseM could degrade HA and various types of CSs and showed almost the same enzyme activity towards CS-A, CS-B, CS-C, CS-D, and HA (100, 99, 98, 99, and 102%), which was different from other conventional hyaluronidases and chondroitinase (Table 3)**.** Moreover, both the *V*_max_ and *K*_m_ showed that HCLaseM was similarly active towards HA and CS-A, and possessed similarly affinity towards HA and CS-A. A phylogenetic tree showed that HCLaseM could not be classified into the existing three subfamilies of the PL-8 family, which also made it possible for it and some other unclassified enzymes to become a new subfamily. Based on these results, the new enzyme was designated as HCLaseM instead of hyaluronidase or chondroitinase. HCLaseM enriched the knowledge of enzymes of the PL-8 family and may have the potential to be an effective tool for preparation of functional oligosaccharides and investigation function relationship of HA and various CSs.

The optimum temperature for the activity of HCLaseM towards both HA and CS-A was 40 °C, which was similar to most enzymes of the PL-8 family (Table 4). HCLaseM kept stable below 30 °C. HCLaseM showed the best activity at pH 7.0, which was similar to most of the previous GAG lyases with an optimum pH of 6.0–8.0 (Table 4). HCLaseM retained about 70% of activity at pH 5.0–9.0, and retained about 90% of activity at pH 6.0–8.0. These properties were beneficial to the storage of the enzyme. Hyaluronidase activity of HCLaseM preserved better than chondroitinase activity in an alkaline environment, which indicates that pH and substrates may affect the stability of HCLaseM.

Hg^2+^, Fe^2+^, and Cu^2+^ strongly inhibited the activity of HCLaseM. The affinity of Hg^2+^, Fe^2+^, and Cu^2+^ for SH, CO, and NH moieties of amino acids leads to structural alterations of enzyme, which inhibits enzyme activity [22]. Except Hg^2+^, Fe^2+^, and Cu^2+^, most metal ions and EDTA did not significantly affect the activity of HCLaseM. The result shows that HCLaseM was not a metal ion-dependent enzyme and was resistant to many metal ions. This property is contributed to application of HCLaseM in complex environment. 

Studies have shown that HA oligosaccharides have important application prospects in the field of medicine [2], such as applications in promotion of angiogenesis [7], immunomodulation, and anti-tumor [38]. Chondroitin sulfate oligosaccharides have antioxidant effects and exert neuroprotective effects to prevent and treat Alzheimer's disease [9]. HCLaseM showed an endogenous mode of action and degraded the substrates to disaccharides. HCLaseM has certain application prospects in the structural and functional studies of glycosaminoglycans and preparation of functional oligosaccharides.

One of the most important fields of application of hyaluronidase is as a drug diffusion agent. The same dose of HCLaseM and commercially available BTH produced similar diffusion-promoting effects. It showed that in a relatively short period of time, the diffusion of trypan blue had nothing to do with the type of enzymes. The diffusion-promoting effect of HCLaseM is dose-dependent, which is beneficial to explore the curative effect of combination therapy with hyaluronidase and drugs. Compared with BTH, HCLaseM is easier to purify and has higher purity. HCLaseM has the potential to become a good drug diffusion agent. Immune problems still need to be considered, and we consider building long-acting HCLaseM to improve application value.

## 4. Materials and Methods 

### 4.1. Material and Animals

*Microbacterium* sp. H14 was isolated from offshore of Qingdao and was preserved in our lab (School of Medicine and Pharmacy, Ocean University of China). Genomic sequencing was performed by Novogene Bioinformatics Technology Co. Ltd. (Beijing, China). PrimeSTAR HS DNA polymerases, restriction endonuclease, T_4_-DNA Ligase Kit, and other genetic engineering enzymes were purchased from Takara Inc. Hyaluronidase from bovine testes (BTH) and HA was purchased from Sigma. CS-A, CS-B, CS-C, and CS-D were obtained by Bomei Biotechnology Co. Ltd. (Hefei, China). Superdex peptide 10/300 GL was purchased from GE Healthcare. *E. coli* DH5α and BL21 (DE3) were grown in Luria-Bertani (LB). Female Kunming mice weighing 18–22 g were purchased from Qingdao Daren Fucheng Co. Ltd. (Qingdao, China). All animal experiments for this research conformed to the rules of international moral principles and the Guidelines for the Care and Use of Laboratory Animals [39]. All animal procedures adhered to compliance according to Animal Ethics Committee of School of Medicine and Pharmacy, Ocean University of China (Qingdao, China; Approval Date: 5 March 2018).

### 4.2. Cloning and Sequence Analysis of GAG Lyase Gene

According to the genomic sequence of *Microbacterium* sp. H14, primers HCLaseM-F (GGAATTCCATATGTTCACCCCCTCTCGCC) and HCLaseM-R (CCGCTCGAGGCGGTGCAGCGAGAACTCC) containing *Nde* I and *Xho* I restriction sites were designed and synthesized. The target gene was amplified using the genomic DNA of *Microbacterium* sp. H14 as a template and ligated into plasmid pET-28a. The recombinant plasmid was sequenced in Beijing Ruibo Xingke Biotechnology Co. Ltd. (Beijing, China). The similarity search of HCLaseM was performed using the BLASTp server of NCBI. The theoretical molecular mass and isoelectric point of the protein was estimated using the Compute pI/Mw tool on the ExPASy server. Phylogenetic analysis was performed using MEGA tools.

### 4.3. Expression and Purification of Recombinant HCLaseM

The recombinant plasmid was transformed to *E. coli* BL21 (DE3) and cultured in LB with kanamycin (30 μg/mL) at 37 °C. The recombinant strains were induced with 0.02 mM IPTG to express recombinant HCLaseM at 18 °C for 24 h when the OD_600_ reached 0.6. Cells were harvested by centrifugation and disrupted by high pressure cracker. The supernatant was obtained by centrifugation and the target protein was purified using an Ni-Sepharose column, and the molecular weight and purity of the target protein were examined by SDS-PAGE. Protein concentration was measured using the BCA protein assay kit (Beyotime Biotechnology, Shanghai, China).

### 4.4. Assay of HCLaseM Activity

First, 0.1 mL of HCLaseM was added to 0.9 mL of 0.2% (*w*/*v*) HA/CS substrate (20 mM PB buffer, pH 7.0), and the enzymatic reaction was carried out at 40 °C for 10 min. HCLaseM forms a double bond in the non-reducing end of the sugar ring by β-elimination mechanism, which can be detected by a change in absorbance at 232 nm. One unit (U) was defined as the amount of protein needed to form 1 µmol of 4, 5-unsaturated uronic acid/min. Millimolar absorption coefficients for HA CS-A, CS-B, CS-C, and CS-D were 5.5, 5.1, 5.1, 5.5, and 5.1 respectively [40].

### 4.5. Substrate Specificity of HCLaseM

HA, CS-A, CS-B, CS-C, and CS-D (0.2% *w*/*v*) were used as substrates to study the substrate specificity of HCLaseM.

### 4.6. Effects of Temperature, pH, Metal Ions, Chelators, and Detergents on HCLaseM

To determine the optimal reaction temperature for HCLaseM activity, HA and CS-A were digested at a range of 0–60 °C, respectively. After incubation at 0–60 °C for 1 h, the residual activity was measured to detect the thermostability. To detect the optimum pH, 0.2% (*w*/*v*) HA substrate was dissolved in different buffers (50 mM Na_2_HPO_4_–NaH_2_PO_4_ (pH 6.0–8.0), Tris-HCl (pH 7.05–8.95), NaH_2_PO_4_–citric acid (pH 3.0–8.0), and glycine–NaOH (pH 8.6–10.6)) with pH 3.0 to 10.0. To determine acidity stability, residual activity was measured after the enzyme was incubated in different buffers at 4 °C for 12 h. Enzyme activity was investigated in the presence of different metal ions, chelators and detergents to detect their effects on HCLaseM.

### 4.7. Enzymatic Reaction Kinetics of HCLaseM Towards HA and CS-A

*K*_m_ and *V*_max_ values of HCLaseM were obtained using Lineweaver–Burk plots. Optimal reaction conditions were used in experiments designed to calculate reaction rate at each substrate concentration to determine *K*_m_ and *V*_max_ values of HCLaseM.

### 4.8. Degradation Pattern of HCLaseM

HA and CS-A were used as substrates to judge the degradation pattern of HCLaseM. First, 1 mL (0.5 U) of enzymes was added to 9 mL of 0.2% (*w*/*v*) HA/CS substrate (20 mM PB buffer, pH 7.0), and incubated at 40 °C. Aliquots of the reaction products were removed for time course experiments and separated on a TLC aluminum silica gel plate developed with *n*-butanol/glacial acetic acid/water (2:1:1, by vol.), and the reaction products were visualized by heating the TLC plate after spraying with a diphenylamine–aniline–phosphate reagent. 

### 4.9. End Products of HCLaseM

First, 2 U of enzymes was added to 2 mL of 0.2% (*w*/*v*) HA and CS-A substrate solution and incubated at 40 °C for 24 h to obtain end products. The final products were separated by a mobile phase (0.2 M ammonium bicarbonate) at a flow rate of 0.2 mL/min using Superdex peptide 10/300 GL gel filtration column, and then analyzed by negative ion electrospray ionization mass spectroscopy (ESI–MS). The analysis was set in the negative ion mode and the mass acquisition range was set at 200–1000.

### 4.10. Effect of Hyaluronidase on the Diffusion of Trypan Blue

Female Kunming mice were selected as the research animals and trypan blue was used as a visual tracer to simulate a combination drug. Hyaluronidase activity was determined according to published recommendations [41]. To investigate the effects of HCLaseM and BTH on the diffusion of trypan blue, mice were randomly divided into three groups with ten cases each group. After mice were thoroughly anesthetized, the median villi of the back were removed and the epidermis was exposed. Trypan blue and test article (50 U BTH, 50 U HCLaseM, PB as negative control) were injected subcutaneously in the median back, with a final volume of 50 μL. The diffusion area at different time points (0, 5, 10, 15, 20, 30, 40, 50 and 60 min) was measured with a caliper after the injection. The trypan blue diffusion area at each time point was calculated according to the area calculation formula (Area = *D*_1_ × *D*_2_ × π/4). *D*_1_ and *D*_2_ represent the lateral diameter and the longitudinal diameter, respectively. To investigate the effect of different concentrations of HCLaseM on the diffusion of trypan blue, mice were randomly divided into four groups, ten cases each group. Trypan blue and different concentrations of HCLaseM (10, 20 and 50 U, PB as negative control) were injected. Subsequent steps are as described above.

## 5. Conclusions

We cloned, expressed, and characterized a new GAG lyase HCLaseM from *Microbacterium* sp. H14. The stability under a wide range of pH, metal ion-resisted property, and substrate specificity of HCLaseM make it a novel enzyme for further basic research and application. The enzyme shows almost the same enzyme activity towards HA, CS-A, CS-B, CS-C, and CS-D, which is different from the reported enzymes and makes HCLaseM a more unique enzyme. HCLaseM may be an effective tool for structural analysis of HA and CS and preparation of oligosaccharides. HCLaseM has the potential to be a good drug diffusion agent.

## Figures and Tables

**Table 1:** Summary of the purified HCLaseM.

Step	Total Activity (U)	Total Protein (mg)	Specific Activity (U/mg Protein)	Fold Purification	Yield (%)
Fermentation media	7671	299.4	25.6	1	100
Nickel column	4290	15.4	278.3	10.9	55.9

0.2% (*w*/*v*) of HA was used as substrate. One unit (U) was defined as the amount of protein needed to form 1 µmol unsaturated uronic acid/min. A millimolar absorption coefficient of 5.5 was used in the calculation.

**Table 2:** *K*_m_ and *V*_max_ values of HCLaseM.

Substrate	*K*_m_ (mg/mL)	*V*_max_ (μM/min)
HA	0.419 ± 0.019	0.0246 ± 0.0003
CS-A	0.478 ± 0.015	0.0264 ± 0.0002

The *K*_m_ and *V*_max_ values of HCLaseM were determined using non-linear regression.

**Table 3:** Comparison of substrate specificity of HCLaseM with glycosaminoglycan lyase from different microorganisms.

Substrate						
Enzyme	Source	CS-A	CS-B	CS-C	HA	Reference
HCLaseM	*Microbacterium* sp. H14	100	99	98	102	This study
ChSase ABC	*Bacteroides* *Stercoris* ^1^	100	32	40	0	[18]
ChSase AC	*B* *. Stercoris* ^1^	100	0	46	67	[18]
ChSase ABC	*Flavobacterium* *Heparinum* ^1^	100	100	100	0	[18]
ChSase AC	*F. Heparinum* ^1^	100	0	110	107	[18]
ChSase ABC	*B.* *Thetaiotaomicron* ^1^	100	13–16	80–130	10–30	[18]
ChSase ABC	*Proteus. Vulgaris* ^1^	100	34	100	60	[18]
BniHL	*Bacillus niacin* ^2^	100	0	68	227	[19]
HAase-B	*Bacillus* sp. A50 ^2^	100	0	36	256	[20]
HCLase	*Vibrio* sp. FC509 ^2^	100	0	96	222	[21]
ChSase ABC	*Acinetobacter* sp. C26 ^2^	100	96	95	133	[22]
ChSase AC II	*Arthrobacter* sp. CS01 ^2^	100	0	86	255	[23]

Activity on CS-A as the substrate was set at 100%. ^1^ Data from the [18]. ^2^ Data was calculated from the specific activity or relative activity in the reference.

**Table 4:** Comparison of the properties of HCLaseM with those of glycosaminoglycan lyase from different microorganisms.

Enzyme	Source	Molecular Mass (kDa)	Optimal pH	Optimal Temperature (°C)	Action Pattern	References
HCLaseM	*Microbacterium* sp. H14	85.9	7	40	Endo	This study
AsChnAC	*Arthrobacter* sp.	N/A	7.2	37	Exo	[15]
ChoA1	*Arthrobacter* sp. MAT3885	83.4	6.0–7.5	40	N/A	[16]
ChSase AC	*Arthrobacter aurescens*	N/A	N/A	N/A	Exo	[17]
ChSase ABC	*Bacteroides stercoris*	116	7	40	Exo	[18]
ChSase AC	*Bacteroides stercoris*	84	5.7–6.0	45–50	Endo	[18]
BniHL	*Bacillus niacin*	120	6	45	Endo	[19]
HAase-B	*Bacillus* sp. A50	116	6.5	44	Endo	[20]
HCLase	*Vibrio* sp. FC509	90	8	30	Endo	[21]
ChSase ABC	*Acinetobacter* sp. C26	76	6	42	N/A	[22]
ChSase AC II	*Arthrobacter* sp. CS01	100	6.5	37	Exo	[23]
cABC I	*Proteus vulgaris*	112	8	37	Endo	[31]
cABC II	*Proteus vulgaris*	105	8	37	Exo	[32]
ChonABC	*Bacteroides thetaiotaomicron*	115	7.6	37	Exo	[33]
ChSase AC	*Flavobacterium heparinum*	74	6.8	40	Endo	[34]
HylB	*Streptococcus zooepidemicus*	N/A	6	37	N/A	[35]
HAase	*Arthrobacter globiformis*	74	6	42	N/A	[36]
ChSase ABC	*Sphingomonas paucimobilis*	82	6.5	40	N/A	[37]

N/A: There are no clear data in the reference.
